# Articular Cartilage Injury of the Knee

**Published:** 2014-01

**Authors:** Fares Haddad

*Articular Cartilage Injury of the Knee* manages to concisely bridge the translational gap from the basic science of articular cartilage and cartilage injury all the way through to current techniques of chondral repair/reconstruction. It is a beautifully illustrated and very nicely presented update on the diagnosis and planning, the basic science and techniques, and the postoperative management and outcomes of modern chondral surgery.

A wide array of authors, notably from North America, Italy and Belgium, have come together to succinctly present the key features of chondral injury in the knee, its characterisation both in terms of imaging and surgery and the potential solutions that are available from a scientific and a clinical perspective. The book covers the state of the art but also allows some room for visionary thinking around the future of functional chondral imaging, the role for new biomarkers and stem cell type interventions. It also quite rightly focuses on rehabilitation and not just on the role of surgery.

The authors have included some high quality radiographs and illustrations. The chapters have been trimmed to make them easily palatable and understood. The references are up to date and appropriate. This book achieves its aims of combining a contemporary summary of the applied basic science, the latest in surgical and implant innovation, new philosophies, techniques and rehabilitation principles. It is an excellent resource for every orthopaedic surgeon but also for any scientist, sports doctor or therapist who is interested in this area or involved in the care of patients with chondral disease.

